# Supplementary material for: Distinguishing new from persistent infections at the strain level using longitudinal genotyping data
Source: bioRxiv. 2025 Feb 8:2025.02.06.636982. Preprint. [Version 1] doi: 10.1101/2025.02.06.636982 (PMC11839113; doi:10.1101/2025.02.06.636982)
Supplement: 1 [file NIHPP2025.02.06.636982v1-supplement-1.pdf]

bioRxiv preprint doi: https://doi.org/10.1101/2025.02.06.636982; this version posted February 8, 2025. The copyright holder for this preprint (which was not certified by peer review) is the author/funder, who has granted bioRxiv a license to display the preprint in perpetuity. It is made available under aCC-BY 4.0 International license.

Supplementary Information

Bayesian model

Incorporating treatments in the Bayesian model

If an individual was treated, the persistence coefficients for previously observed alleles are returned to 0, but two treatment coefficients are added for those alleles. The first is an acute coefficient set to 1 until the end of the acute treatment phase (default 10 days), and the second is a longitudinal coefficient set to 1 until the next infection with that allele. This captures the intuition that treatment likely clears previous infections (hence setting the other persistence coefficients to 0) but might not (hence the treatment coefficients set to 1).

Solving for Bayesian model parameters

The following result will be necessary for solving for $p_{Y_{ita}}$. Suppose A_1, B_1, A_2, B_2, C are events such that C happens if A_2 or B_2 happen, A_2 happens with probability a_2 given A_1 (and 0 otherwise), B_2 happens independently with probability b_2 given B_1 (and 0 otherwise), and A_1 and B_1 happen independently with probability a_1 and b_1 respectively. The probability of interest is $P(C|A_1 \cup B_1)$.

$$
\begin{aligned}
P(C|A_1 \cup B_1) &= \frac{P((A_2 \cup B_2) \cap (A_1 \cup B_1))}{P(A_1 \cup B_1)} \text{ because } C = (A_2 \cup B_2) \\
&= \frac{P((A_2 \cap (A_1 \cup B_1)) \cup (B_2 \cap (A_1 \cup B_1)))}{P(A_1 \cup B_1)} \\
&= \frac{P((A_2 \cap A_1) \cup (B_2 \cap B_1))}{P(A_1 \cup B_1)} \text{ because } A_2 \text{ only if } A_1 \text{ and } B_2 \text{ only if } B_1 \\
&= \frac{P(A_2 \cap A_1) + P(B_2 \cap B_1) - P(A_2 \cap A_1 \cap B_2 \cap B_1)}{P(A_1) + P(B_1) - P(A_1 \cap B_1)} \\
&= \frac{P(A_2|A_1)P(A_1) + P(B_2|B_1)P(B_1) - P(A_2|A_1)P(A_1)P(B_2|B_1)P(B_1)}{P(A_1) + P(B_1) - P(A_1)P(B_1)} \\
&\qquad \text{by independence properties} \\
&= \frac{a_2 a_1 + b_2 b_1 - a_2 a_1 b_2 b_1}{a_1 + b_1 - a_1 b_1}
\end{aligned}
$$

Now, set $Y_{ita} = 1$ as C, A_2 and B_2 as $W^{(0)}_{Y_{ita}} = 1$ and $W^{(1)}_{Y_{ita}} = 1$ respectively, and A_1 and B_1 as $W^{(0)}_{Z_{it}} = 1$ and $W^{(1)}_{Z_{it}} = 1$ respectively. Using the above,

$$
p_{Y_{ita}} = \frac{p_{W^{(1)}_{Y_{ita}}}(1) p_{W^{(1)}_{Z_{it}}} + p_{W^{(0)}_{Y_{ita}}}(1) p_{W^{(0)}_{Z_{it}}} - p_{W^{(1)}_{Y_{ita}}}(1) p_{W^{(1)}_{Z_{it}}} p_{W^{(0)}_{Y_{ita}}}(1) p_{W^{(0)}_{Z_{it}}}}{p_{W^{(1)}_{Z_{it}}} + p_{W^{(0)}_{Z_{it}}} - p_{W^{(1)}_{Z_{it}}} p_{W^{(0)}_{Z_{it}}}}.
$$

The probability that an allele is new given that it is observed is given by:

bioRxiv preprint doi: https://doi.org/10.1101/2025.02.06.636982; this version posted February 8, 2025. The copyright holder for this preprint (which was not certified by peer review) is the author/funder, who has granted bioRxiv a license to display the preprint in perpetuity. It is made available under aCC-BY 4.0 International license.

$$\begin{aligned}
P(W^{(1)}_{Y_{ita}} = 1 | Y_{ita} = 1, \mathbf{X}_{ita} = \mathbf{x}_{ita}) &= P(W^{(1)}_{Y_{ita}} = 1 | Y_{ita} = 1, Z_{it} = 1, \mathbf{X}_{ita} = \mathbf{x}_{ita}) \\
&= \frac{P(W^{(1)}_{Y_{ita}} = 1 | Z_{it} = 1, \mathbf{X}_{ita} = \mathbf{x}_{ita})}{P(Y_{ita} = 1 | Z_{it} = 1, \mathbf{X}_{ita} = \mathbf{x}_{ita})} \\
&= \frac{p_{W^{(1)}_{Y_{ita}}}(1) p_{W^{(1)}_{Z_{it}}}}{p_{W^{(1)}_{Y_{ita}}}(1) p_{W^{(1)}_{Z_{it}}} + p_{W^{(0)}_{Y_{ita}}}(1) p_{W^{(0)}_{Z_{it}}} - p_{W^{(1)}_{Y_{ita}}}(1) p_{W^{(1)}_{Z_{it}}} p_{W^{(0)}_{Y_{ita}}}(1) p_{W^{(0)}_{Z_{it}}}}
\end{aligned}$$

Bayesian model with major drop-out

If there is major sequencing drop-out, a model can be used that allows alleles that have never been observed before to be called persistent:

$$\log\left(\frac{p_{W^{(0)}_{Y_{ita}}}(w)}{1 - p_{W^{(0)}_{Y_{ita}}}(w)}\right) = \begin{cases} \sum_{j \in \text{Persistence covariates}} \beta_{j,a} x_{jita} & \text{if any } x_{jit} > 0 \text{ and } w = 1 \\ \delta_a \cdot \alpha_{\text{new},0,a} & \text{otherwise, if } w = 1 \\ -\infty & \text{otherwise} \end{cases}$$
$$\text{Subject to: } 0 \le \delta_a \le 1$$

Second, rather than only considering the Y_{ita} such that $Z_{it} = 1$, all Y_{ita} are modeled, unconditional on $Z_{it} = 1$ since the $Z_{it} = 1$ are less accurate:

$$\begin{aligned}
P(Y_{ita} = 1 | \mathbf{X}_{ita} = \mathbf{x}_{ita}) = P(W^{(1)}_{Y_{ita}} = 1 | \mathbf{X}_{ita} = \mathbf{x}_{ita}) &+ P(W^{(0)}_{Y_{ita}} = 1 | \mathbf{X}_{ita} = \mathbf{x}_{ita}) \\
&- P(W^{(1)}_{Y_{ita}} = 1 | \mathbf{X}_{ita} = \mathbf{x}_{ita}) P(W^{(0)}_{Y_{ita}} = 1 | \mathbf{X}_{ita} = \mathbf{x}_{ita}) \\
= p_{W^{(1)}_{Y_{ita}}}(1) p_{W^{(1)}_{Z_{it}}} &+ p_{W^{(0)}_{Y_{ita}}}(1) p_{W^{(0)}_{Z_{it}}} - p_{W^{(1)}_{Y_{ita}}}(1) p_{W^{(1)}_{Z_{it}}} p_{W^{(0)}_{Y_{ita}}}(1) p_{W^{(0)}_{Z_{it}}}
\end{aligned}$$

The rest of the model remains the same. In practice, this exposes the second step to many more instances in which $Y_{ita} = 0$, leading to lower values for all the parameters and particularly those for new infections.

Bayesian model priors

$$\begin{aligned}
\mu_{\alpha,\text{alleles new}} &\sim \mathcal{N}(-3, 10) \\
\sigma_{\alpha,\text{alleles new}} &\sim \text{Cauchy}_{\ge 0}(0, 10) \\
\mu_{\text{alleles old}} &\sim \mathcal{N}(0, 10) \\
\sigma_{\text{alleles old}} &\sim \text{Cauchy}_{\ge 0}(0, 10) \\
\alpha_0 &\sim \mathcal{N}(-3, 10) \\
\beta_j &\sim \mathcal{N}(0, 10), \text{with the constraint } \beta_j \ge 0 \text{ for } j \in \text{persistence covariates} \\
\alpha_{\text{new},0,a} &\sim \mathcal{N}(\mu_{\alpha,\text{alleles new}}, \sigma_{\alpha,\text{alleles new}}) \\
\beta_{j,a} &\sim \mathcal{N}(\mu_{\text{alleles old}}, \sigma_{\text{alleles old}})
\end{aligned}$$

Bayesian model specification with major drop-out

When using the drop-out option, the model is augmented by the following:

bioRxiv preprint doi: https://doi.org/10.1101/2025.02.06.636982; this version posted February 8, 2025. The copyright holder for this preprint (which was not certified by peer review) is the author/funder, who has granted bioRxiv a license to display the preprint in perpetuity. It is made available under aCC-BY 4.0 International license.

$$
\begin{aligned}
a_{\text{drop out}} &\sim \text{Expo}(1) \\
b_{\text{drop out}} &\sim \text{Expo}(1) \\
\delta_a &\sim \text{Beta}(a_{\text{drop out}}, b_{\text{drop out}})
\end{aligned}
$$

Unequal intervals between visits

Because the probability of a new infection in the Bayesian model depends only on the allele and the covariates, shorter times between appointments do not directly result in lower probabilities of new infections. The model could be modified to consider this interval length, but doing so risks inflating the apparent new infection rate if sequencing drop-out at very close time points results in alleles that appear new despite very small intervals. Additionally, this can substantially increase computational complexity because the terms in the likelihood cannot be compressed as effectively when there are many unique interval lengths. If unequal intervals are of concern, general covariates can be included to indicate whether the time point follows e.g. a very short (≤ 2 days), short ($3-7$ days), medium ($8-21$ days), or long (≥ 22 days) interval. This would allow different probabilities of a new infection following each interval, but it could increase variability if there are few intervals of particular lengths.

Clustering model

Probability new calculation

Consider a single subject, and suppose there are T time points, A alleles, and N_C clusters of alleles $C_1, ..., C_{N_C}$ with each cluster being a subset of total alleles ($\subseteq \{1, ..., A\}$). Let p_{t,C_j} be the probability cluster C_j is present at time point t, and let p_{a,C_j} be the probability allele a is in cluster C_j. Assume these have already been determined from the likelihood maximization step. Then, assuming the clusters are independent of each other,

$$
\begin{aligned}
P(a \text{ present at time } t) &= 1 - \prod_{j=1}^{N_c} (1 - p_{a,C_j} p_{t,C_j}) \\
p_{a \in C_j, t|a,t} &= P(a \in C_j \text{ at time } t | a \text{ present at time } t) \\
&= \frac{p_{a,C_j} p_{t,C_j}}{1 - \prod_{j=1}^{N_c} (1 - p_{a,C_j} p_{t,C_j})} \\
p_{a \notin C_j, t} &= P(a \notin C_j \text{ before time } t) \\
&= \begin{cases} 1 \text{ if there is no } t' \leq t \text{ such that } a \text{ is present at } t' \\ \prod_{t' \leq t \text{ such that } a \text{ is present at } t'} (1 - p_{a \in C_j, t'|a,t'}) \text{ otherwise} \end{cases} \\
P(a \text{ present at time } t \text{ and new}) &= 1 - \prod_{j=1}^{N_c} ((1 - p_{a \notin C_j, t} p_{a,C_j}) p_{t,C_j} + (1 - p_{t,C_j})) \\
P(a \text{ new at } t | a \text{ present at } t) &= \frac{P(a \text{ present at time } t \text{ and new})}{P(a \text{ present at time } t)}
\end{aligned}
$$

Multiple imputation criteria

A proposed allele based on the sampling scheme described earlier is only allowed if it satisfies at least one of the following criteria:

bioRxiv preprint doi: https://doi.org/10.1101/2025.02.06.636982; this version posted February 8, 2025. The copyright holder for this preprint (which was not certified by peer review) is the author/funder, who has granted bioRxiv a license to display the preprint in perpetuity. It is made available under aCC-BY 4.0 International license.

1. No arbitrary singletons: the allele has been sequenced or imputed at some other time point for the subject.
2. No spurious infections: the allele is first imputed at the same time point as a second allele is first imputed such that the second allele is sequenced at some time point after being imputed (i.e., the second is not an arbitrary singleton).
3. No neighbors: There are no alleles sequenced within $k_{\text{max}}/2$ time points before or after the current time point.

The no arbitrary singletons and no spurious infections rules together ensure that if there is an infection at a time point with only pathogen positivity, the estimated molFOI remains representatively high without introducing spurious new infection events. The no neighbors rule ensures that if there is effectively no information regarding what allele should be imputed, *some* allele is still imputed to show an infection.

Additionally, imputed datasets are only accepted if all time points with missingness are imputed to have at least one allele present. If this is not the case, the imputation is retried until this is the case or until a retry limit is reached (default 200). If the retry limit is reached, time points are imputed with the closest sequenced allele if the closest is within $k_{\text{max}}/2$ time points and a random allele otherwise.

Synthetic evaluation data generation

For both synthetic data types (rolling presence probability and Poisson time to clearance), new infections were generated as follows.

1. For each allele (default $n_{\text{alleles}} = 100$), a new infection probability is generated from a Beta(1/infection multiplier, 50) distribution (default infection multiplier 5).
2. For each subject (default 200 subjects), time points representing appointments (the number of which is sampled uniformly from 7 to 12) are evenly spaced from days 1 to 200.
3. Depending on the model specified, a season indicator is set to 1 for the second half of the time points; a fixed covariate indicator is set to 1 for each subject with probability 1/2; a time-varying covariate is set to 1 at each time with probability 1/2; and/or a prevention covariate indicator is set to 1 for each subject with probability 1/2.
4. Multiplicative modifications to the new infection probabilities are made by (1) sampling from a length n_{alleles} multivariate normal centered at 0 with variance 1 and covariance 0.4 (season), variance 0.5 and covariance 0.25 (fixed covariate), or variance 0.5 and covariance 0.15 (time varying covariate); (2) multiplying these modifiers by their indicators; and (3) exponentiating these products and multiplying them by the new infection probabilities.
5. For each subject, at each time point, new infections are sampled as independent Bernoulli random variables with the modified probabilities above. If the number of new alleles is nonzero, a number of additional alleles to be present is sampled from a Poisson with rate parameter equal to the number of new alleles (1) multiplied by the infection multiplier and (2) multiplied by 1/2 if the prevention covariate is 1. Then, that many additional alleles are sampled with probabilities proportional to their modified new infection probabilities.

Finally, new infections are not allowed at neighboring time points since these would be

bioRxiv preprint doi: https://doi.org/10.1101/2025.02.06.636982; this version posted February 8, 2025. The copyright holder for this preprint (which was not certified by peer review) is the author/funder, who has granted bioRxiv a license to display the preprint in perpetuity. It is made available under aCC-BY 4.0 International license.

effectively indistinguishable from sequencing drop out in real data. Instead, to simulate sequencing drop-out, with a probability given by the drop-out parameter (default 0.2), each new allele is removed and placed at the next time point as new.

The simulation of persistent alleles differs between the rolling presence probability and Poisson time to clearance models. For the rolling presence probability model, a persistence probability of presence is drawn from a Beta$(1,5)$ distribution for each allele. Likewise, a lag probability of presence is drawn from a Beta$(1,1)$ distribution for each allele. Then, on time points after the first infection with an allele, the allele is marked present and persistent according to its persistence probability. If the allele has been observed in the last 30 days, the allele is marked as present and persistent according to its lag probability.

For the models involving treatment, if the subject is infected at the time point, the subject is treated with probability 0.1, and the treatment applies to each allele with a probability of 0.9. If the treatment applies to an allele, the subject is considered to have not had infections from that allele for the purpose of generating persistent infections at subsequent time points. This is intended to simulate treatment that is mostly, but not completely, effective.

For the Poisson time to clearance model, when a set of alleles is new, the number of time points to clearance (for all those alleles) is drawn from a Poisson distribution with rate 2 (80% probability) or a rate equal to the total number of time points (20% probability). These are chosen to simulate short- and long-term infections similar to the lag and persistent infections in the rolling presence probability model.

When there are multiple loci, the alleles are divided evenly among the loci, one locus is chosen as the primary locus at each time point, new alleles are sampled as above at the primary locus, and alleles at each other locus are chosen with one allele per allele at the primary locus (possibly non-unique). In this way, the most diverse locus is selected, and alleles at the other loci are chosen as though there are underlying non-clonal pathogens with particular allele patterns. Then, the Poisson time to clearance model is applied as before.

Finally, for both simulation strategies, time points with sequenced infections are converted to qRT-PCR positive only time points according to the qPCR probability (default 0).

Synthetic evaluations for robustness

To test the robustness of these results, the number of subjects per datasets, the number of alleles sequenced, and the model properties affecting the rate of new infections were varied. When varying the number of subjects per dataset from 50 to 1000, the accuracy of each model was unchanged (**Sup. Fig. 2**). When varying the number of alleles (but leaving the per-allele infection rates unchanged), the predicted probabilities were mostly unchanged, but the clustering and simple models more severely underestimated the molFOI at high allele counts as increasingly complex infections blurred the distinction between infections. All models underestimated the true number of infections at 200 alleles, but the rate of new infections simulated in that scenario (average 7.5 in 200 days) is likely higher than would be observed in most real datasets. When varying how new infections were generated to account for seasonality, a lack thereof, or seasonality plus a subject-specific protection covariate (e.g., a vaccine or chemoprevention), the accuracy of the models was mostly unchanged. The only infection dynamic to substantially affect accuracy was treatment of subjects for their infections (**Sup. Fig. 2C**). This caused the simple and clustering models to underestimate the probability an allele was from a new infection since these models do not account for treatments which generally eliminate pre-existing infections. It also caused the Bayesian

bioRxiv preprint doi: https://doi.org/10.1101/2025.02.06.636982; this version posted February 8, 2025. The copyright holder for this preprint (which was not certified by peer review) is the author/funder, who has granted bioRxiv a license to display the preprint in perpetuity. It is made available under aCC-BY 4.0 International license.

model to overestimate the number of new infections by 0.97 on average (18% above the average 5.4 infections in this high-transmission simulation) despite not affecting the model's accuracy in determining probabilities or molFOI. Regarding the absolute error, the Bayesian model produced more accurate probabilities and molFOI estimates across all settings, but the clustering model was the most accurate at estimating the number of new infection events for simulations with either low allele counts (20 and 50) or treatment for infections (**Sup. Fig. 3**). Overall, the Bayesian model typically produced the most accurate results and was robust to variations in the underlying data.

Sequence data generation from blood spots

Each of the three genotyping datasets obtained for this study (from the CIS43LS cohort,[16] the 2011 Malian cohort,[9] and the Ugandan cohort[5]) were generated by applying sequential (PCR1 and PCR2) amplification reactions to DNA extracted from dried blood spots collected in Mali and Uganda. In all cases, amplification targeted short *P. falciparum* antigen fragments (188-320 bp, excluding primer binding sites), and sequencing of PCR2-indexed sample pools occurred on an Illumina MiSeq instrument.

For the CIS43LS cohort, PCR amplification involved a multiplex of 6 fragments within the genes CSP (PF37_0304600), TRAP (PF3D7_1335900), SERA8 (PF3D7_0207300), SURFIN (PF3D7_0424400), KELT (PF3D7_1475900), and WD-repeat containing protein (PF3D7_1410300). PCR1 consisted of an initial incubation step at 95°C (3 min); 29 amplification cycles at 98°C (20 s), 57°C (15 s), and 72°C (30 s); and a final extension step at 72°C (1 min). Reaction products subsequently underwent an Exonuclease I digestion and dilution step. PCR2 consisted of an initial incubation step at 95°C (3 min); 10 amplification cycles at 98°C (20 s), 65°C (30s), and 72°C (30 s); and a final extension step at 72°C (1 min). Microhaplotypes were resolved from sequence output by running the malaria amplicon processing pipeline available at https://github.com/broadinstitute/malaria-amplicon-pipeline.git. Alleles were discarded from a sample if represented by < 5 read-pairs in the sample or $< 1\%$ of the total read depth within the sample locus. Alleles which never occurred as major alleles (i.e., never represented the majority of read-pairs within any sample locus) were also excluded from final calls.

For the 2011 Malian cohort,[9] PCR amplification involved a multiplex of 4 antigen fragments within the genes AMA1 (PF3D7_1133400), SERA2 (PF3D7_0207900), CSP (PF37_0304600), and TRAP (PF3D7_1335900). The latter CSP and TRAP targets are identical to those contained within the six-plex used for the CIS43LS cohort. The PCR protocol used for the 2011 Malian cohort, detailed extensively previously,[26] is highly similar to that used for the CIS43LS cohort, although it does not involve an intermediate digestion/dilution step. Initial denoising was performed with the same malaria amplicon processing pipeline. Alleles were discarded from a sample if represented by < 10 read-pairs in the sample or $< 1\%$ of the total read depth within the sample locus. Singleton microhaplotypes (i.e., detection in only a single visit) were also excluded from final calls.

For the Ugandan cohort,[5] PCR amplification involved only a single antigen fragment—the same AMA1 (PF3D7_1133400) target contained within the four-plex used for the 2011 Malian cohort. The PCR protocol used for the Ugandan cohort, previously described,[38] is slightly distinct in that it uses hemi-nested PCR2 primers to increase sensitivity of the reaction. Also unlike in the CIS43LS and 2011 Malian cohorts, the Uganda workflow processed duplicate samples for each visit, and sequencing output was denoised using SeekDeep methods.[39]

bioRxiv preprint doi: https://doi.org/10.1101/2025.02.06.636982; this version posted February 8, 2025. The copyright holder for this preprint (which was not certified by peer review) is the author/funder, who has granted bioRxiv a license to display the preprint in perpetuity. It is made available under aCC-BY 4.0 International license.

805 # Supplementary Figures

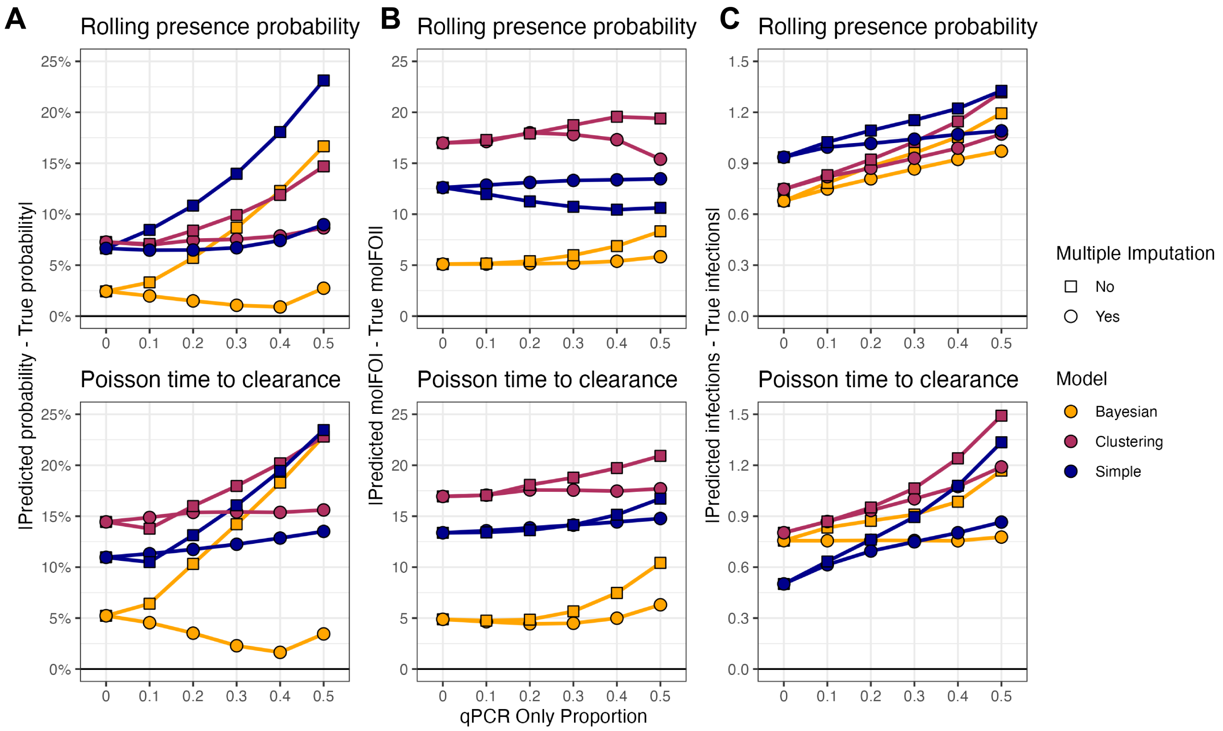

Supplementary Figure 1. The Bayesian model with multiple imputation typically minimizes the absolute error for the probability an individual sequenced allele was new (A), the per-subject molFOI (B), and the per-subject number of infection events (C). Datasets were generated and error metrics were evaluated as in **Fig. 2**.

bioRxiv preprint doi: https://doi.org/10.1101/2025.02.06.636982; this version posted February 8, 2025. The copyright holder for this preprint (which was not certified by peer review) is the author/funder, who has granted bioRxiv a license to display the preprint in perpetuity. It is made available under aCC-BY 4.0 International license.

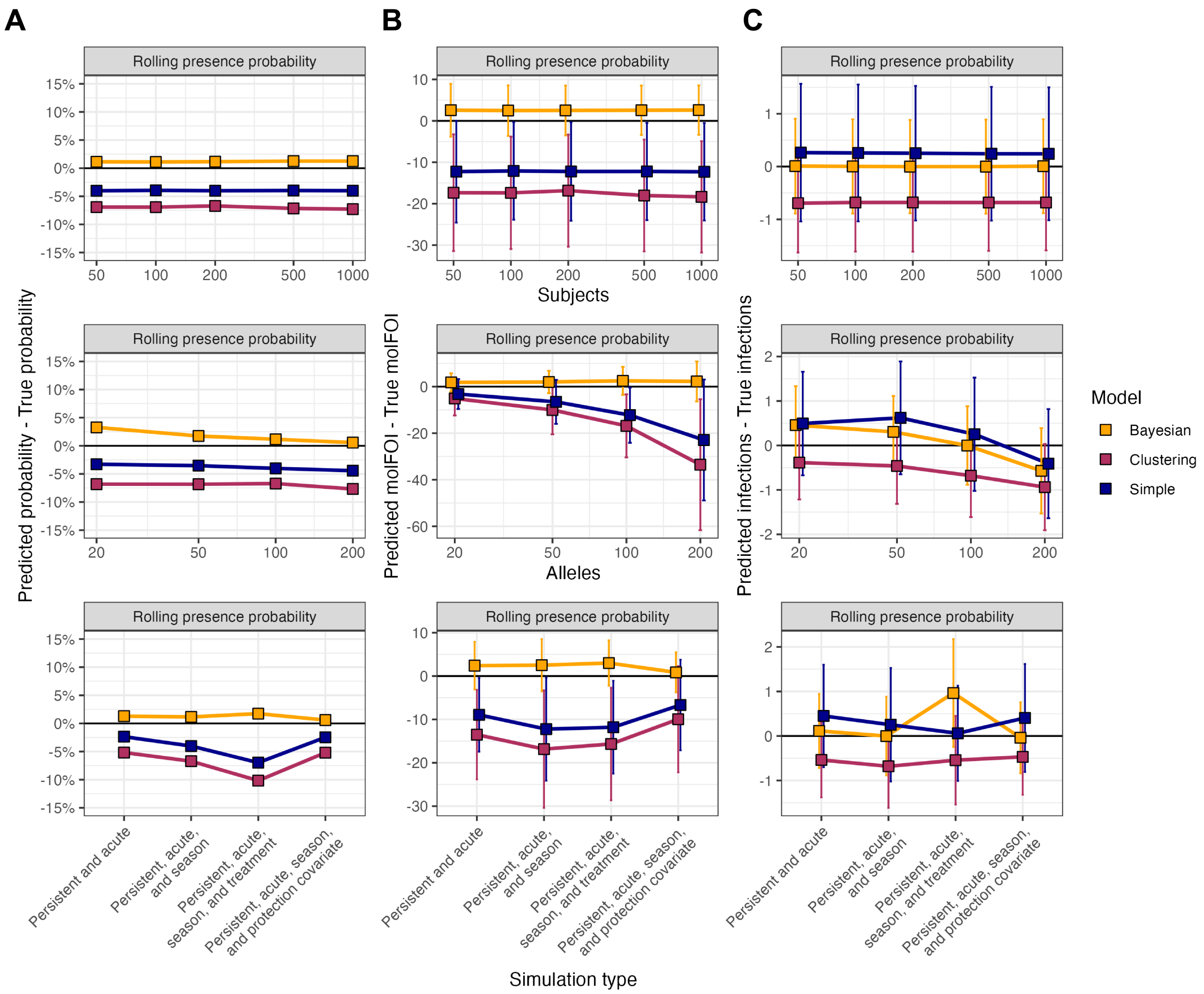

Supplementary Figure 2. Across different numbers of subjects, different numbers of alleles, and different infection models, the Bayesian model typically produces the least biased estimates of the probability an individual sequenced allele was new (A), the per-subject molFOI (B), and the per-subject number of infection events (C). Datasets were generated and error metrics were evaluated as in **Fig. 2** except with no missing (qPCR only) samples and varying numbers of subjects, varying numbers of alleles, or varying models described in the **Supplementary Information**.

bioRxiv preprint doi: https://doi.org/10.1101/2025.02.06.636982; this version posted February 8, 2025. The copyright holder for this preprint (which was not certified by peer review) is the author/funder, who has granted bioRxiv a license to display the preprint in perpetuity. It is made available under aCC-BY 4.0 International license.

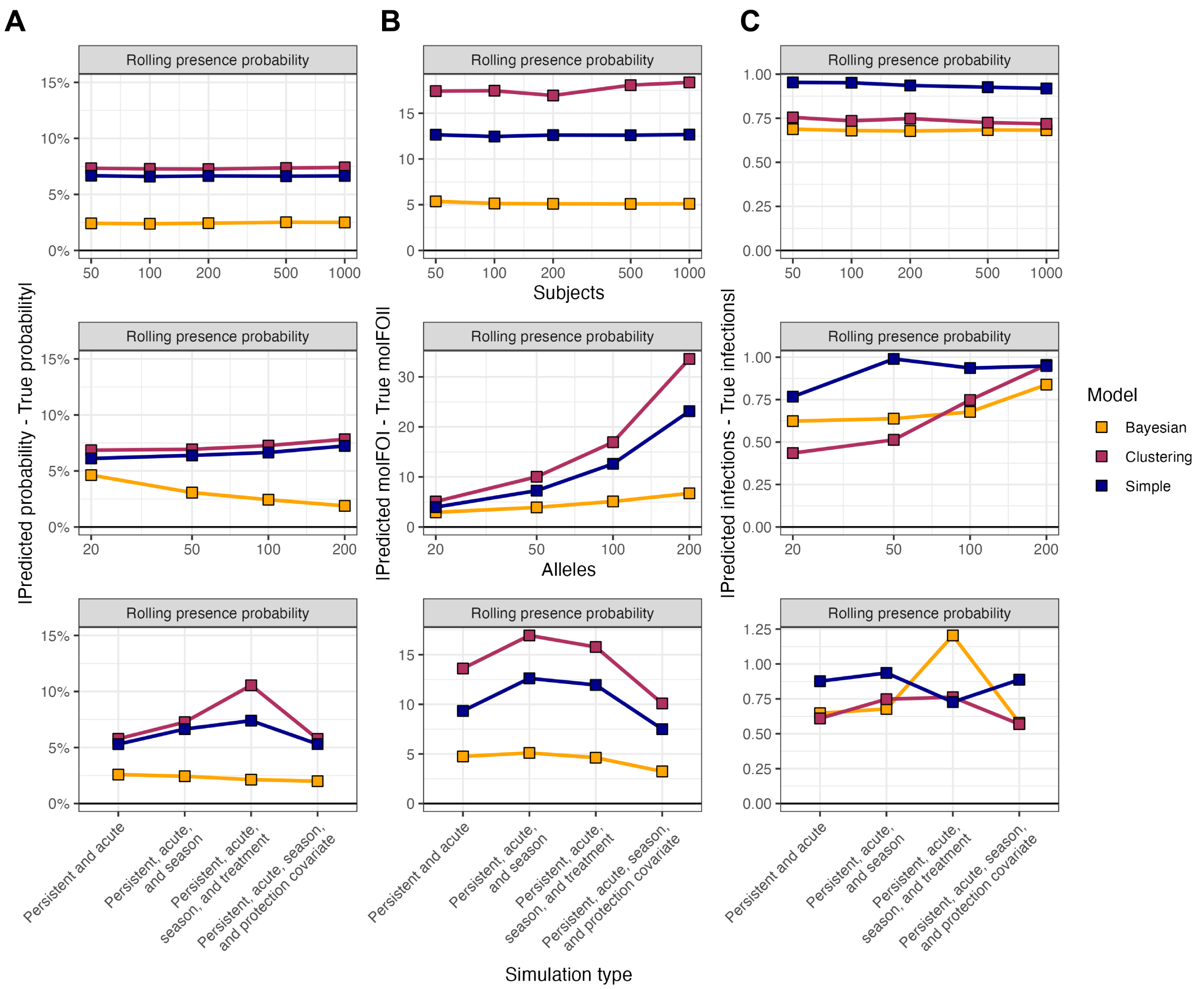

Supplementary Figure 3. Across different numbers of subjects, different numbers of alleles, and different infection models, the Bayesian model typically produces the lowest absolute error in estimating the probability an individual sequenced allele was new (A), the per-subject molFOI (B), and the per-subject number of infection events (C). Datasets were generated and error metrics were evaluated as in **Fig. 2** except with no missing (qPCR only) samples and varying numbers of subjects, varying numbers of alleles, or varying models described in the **Supplementary Information**.

bioRxiv preprint doi: https://doi.org/10.1101/2025.02.06.636982; this version posted February 8, 2025. The copyright holder for this preprint (which was not certified by peer review) is the author/funder, who has granted bioRxiv a license to display the preprint in perpetuity. It is made available under aCC-BY 4.0 International license.

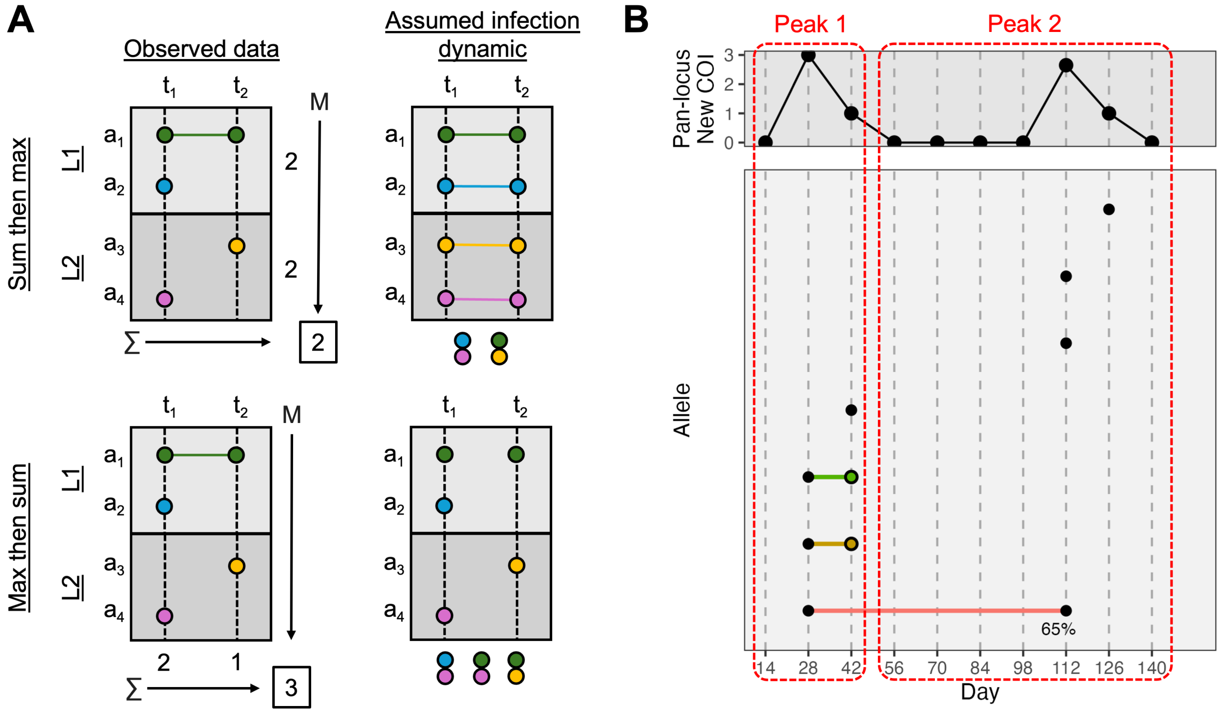

Supplementary Figure 4. Aggregation methods compute epidemiological endpoints of interest. A. For an example time series with four sequenced alleles across two loci, the sum then max and max then sum methods estimate different molFOI values. In the observed data, all alleles are newly observed except a_1 at t_2, which is estimated to be persistent before aggregation. First taking the sum of new alleles for each locus and then taking the maximum of the sums gives a molFOI of 2 while taking the maximum of the number of new alleles per locus per time point and then summing over the time points gives a molFOI of 3. The assumed infection dynamic that would allow each model to be correct is shown to the right. The sum then max model correctly yields a molFOI of 2 when two strains were present at both time points but were sporatically unobserved due to sequencing drop-out. The max then sum model correctly yields a molFOI of 3 when three strains were present at only one time point each and alleles a_1 and a_4 were present in two strains each. For this panel of this figure only, horizontal lines indicate the allele is present due to the same strain persistent at the connected times. **B.** For an example time series, the new infections are estimated. For this panel of this figure only, the solid dots are alleles new with 100% probability unless otherwise labeled, and the open dots are alleles new with 0% probability (i.e., persistent). The time series is divided into two peaks of pan-locus new alleles, each of which contains a time point with an allele with a 100% probability of being new (days 28 and 112), yielding an initial count of 2 new infections. Additionally, the minimum probability an allele is new from each other time point is added. This adds zero at day 42 since the green and yellow alleles have probability zero of being new but adds 1 at day 126 since the top allele is the only one present and has a 100% probability of being new. Thus, the estimated new infection count is 3.

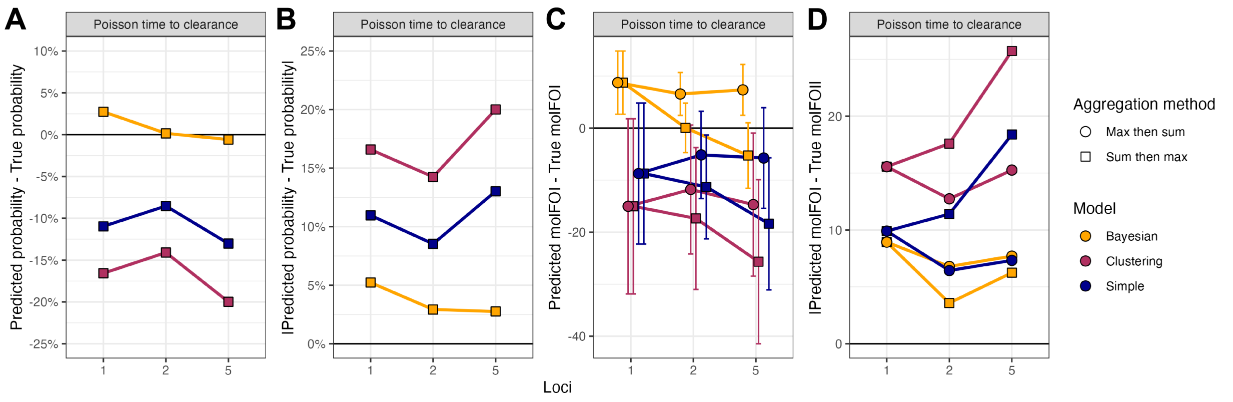

Supplementary Figure 5. Across different numbers of loci sequenced, the Bayesian model typically produces the least biased and lowest absolute error estimates of the probability an individual sequenced allele was new and the per-subject total molFOI. Datasets were generated and error metrics were evaluated as in **Fig. 2** except with no missing (qPCR only) samples and varying numbers of loci with alleles generated as described in the **Supplementary Information**. For computing the molFOI, both the "max then sum" and "sum then max" strategies were applied. New infections were not evaluated because alleles across loci are treated the same as alleles within a locus for determining new infections.

bioRxiv preprint doi: https://doi.org/10.1101/2025.02.06.636982; this version posted February 8, 2025. The copyright holder for this preprint (which was not certified by peer review) is the author/funder, who has granted bioRxiv a license to display the preprint in perpetuity. It is made available under aCC-BY 4.0 International license.

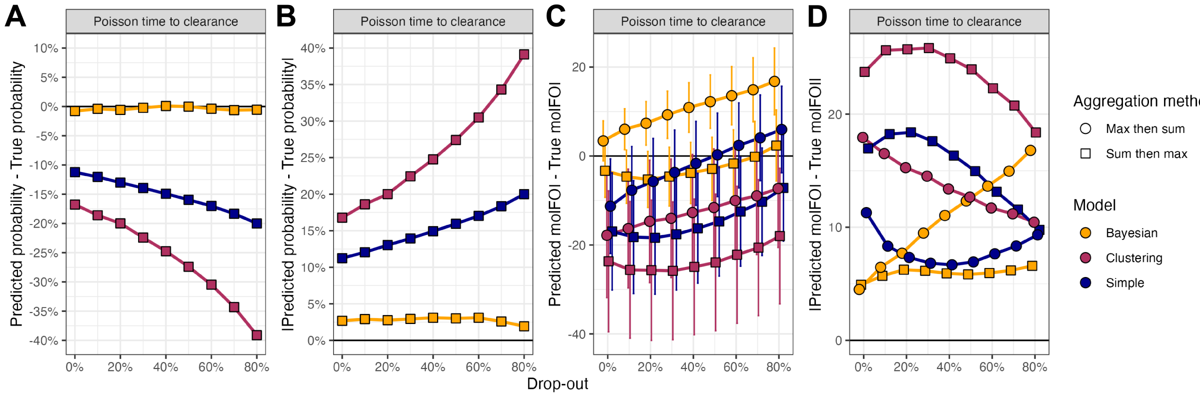

Supplementary Figure 6. Across different rates of sequencing drop-out at the first time point of an infection, the Bayesian model typically produces the least biased and lowest absolute error estimates of the probability an individual sequenced allele was new and the per-subject molFOI. Datasets were generated and error metrics were evaluated as in **Fig. 2** except with no missing (qPCR only) samples and five loci with alleles generated as described in the **Supplementary Information**. For computing the molFOI, both the "max then sum" and "sum then max" strategies were applied. New infections were not evaluated because alleles across loci are treated the same as alleles within a locus for determining new infections.

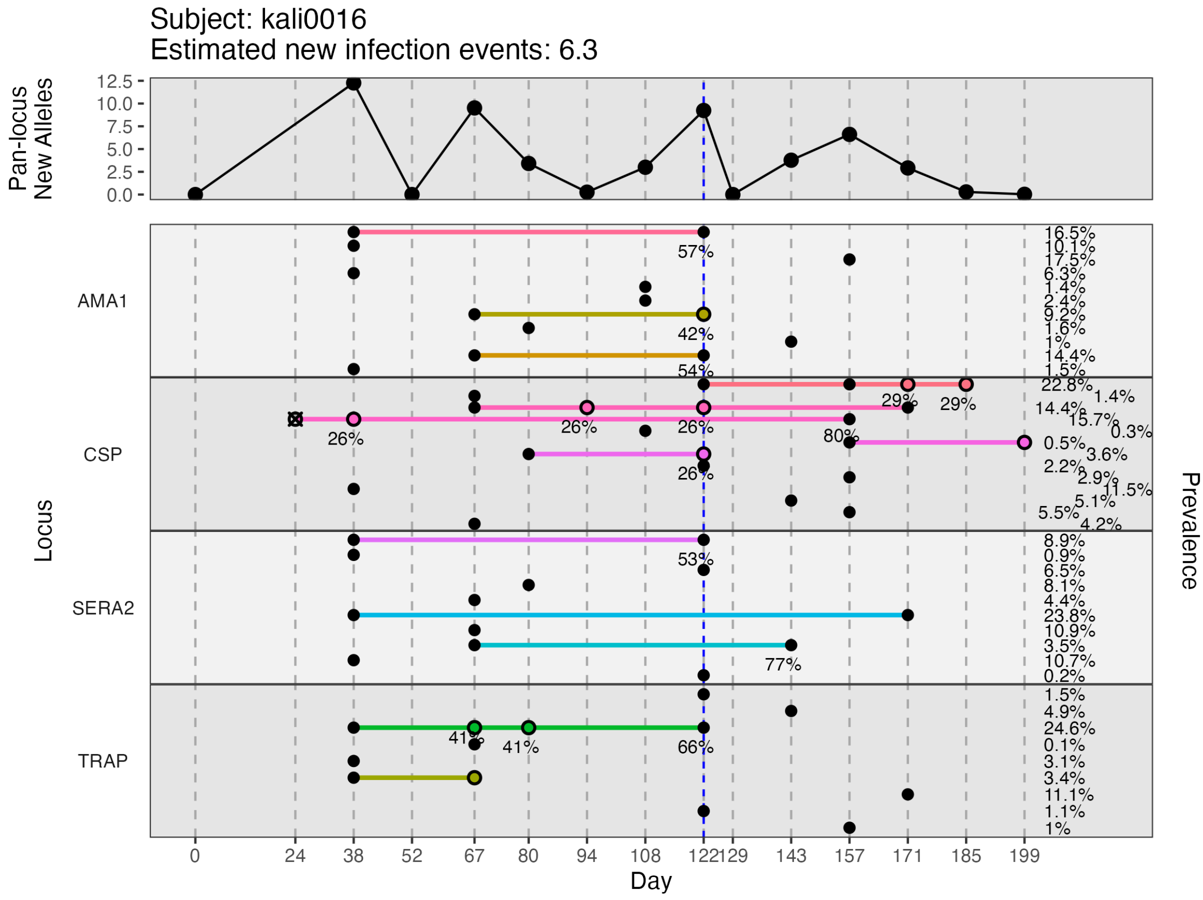

Supplementary Figure 7. A representative example of longitudinal data from an individual in the 2011 Malian cohort assigned probabilities with the Bayesian model. The default DINEMITES vizualization shows the infection course for one individual with one horizontal pane per locus and one row per allele. Vertical dashed lines mark days when the subject provided blood spots, and dots indicate alleles present in genotyping, connected horizontally if observed repeatedly. Treatments for malaria are marked with blue dashes. Probabilities that the alleles were from new infections were assigned using the Bayesian model with parameters for seasonality, treatment, and previous infections ever and in the previous 30, 60, and 90 days. Sequenced alleles are marked with solid circles if their average assigned probabilities of being new are over 50%, and they are marked with open circles otherwise. The probability of the allele being new is only displayed under the point if the probability is between 20% and 80%. The pan-locus new alleles is the sum of the probabilities over all alleles at each time point. The prevalence on the right is the proportion of sequenced infections in which the allele is present.

bioRxiv preprint doi: https://doi.org/10.1101/2025.02.06.636982; this version posted February 8, 2025. The copyright holder for this preprint (which was not certified by peer review) is the author/funder, who has granted bioRxiv a license to display the preprint in perpetuity. It is made available under aCC-BY 4.0 International license.

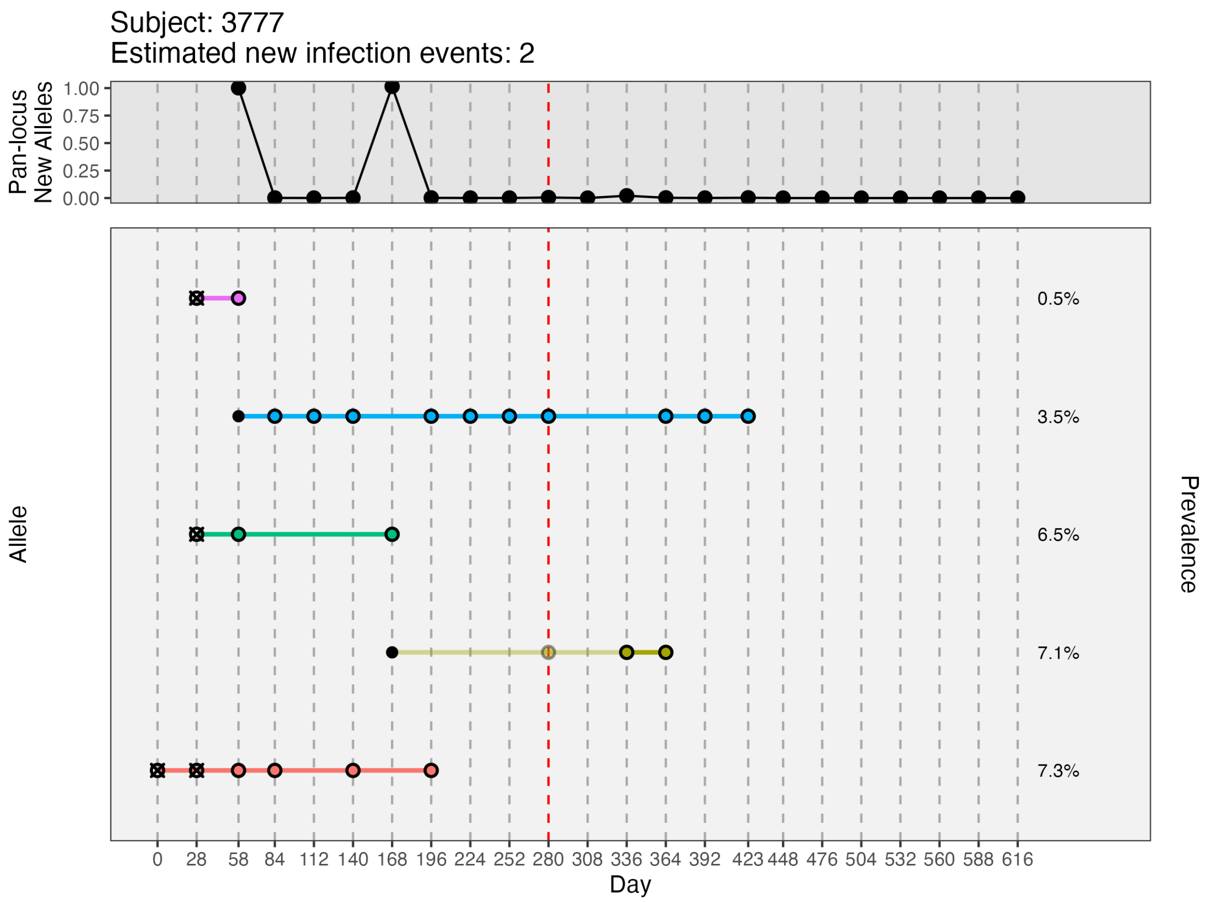

Supplementary Figure 8. A representative example of longitudinal data from an individual in the Ugandan cohort assigned probabilities with the Bayesian model. The default DINEMITES vizualization shows the infection course for one individual with one horizontal pane per locus and one row per allele. Vertical dashed lines mark days when the subject provided blood spots, and dots indicate alleles present in genotyping, connected horizontally if observed repeatedly. Fifty imputed datasets were used, and alleles at missing time points (red dashes) are opaque proportional to their probability of being present as determined in the imputation process. Probabilities that the alleles were from new infections were assigned using the Bayesian model with parameters for seasonality, treatment, and previous infections ever and in the previous 30, 60, and 90 days. Sequenced alleles are marked with solid circles if their average assigned probabilities of being new are over 50%, and they are marked with open circles otherwise. The probability of the allele being new is only displayed under the point if the probability is between 20% and 80%. The pan-locus new alleles is the sum of the probabilities over all alleles at each time point. The prevalence on the right is the proportion of sequenced infections in which the allele is present.

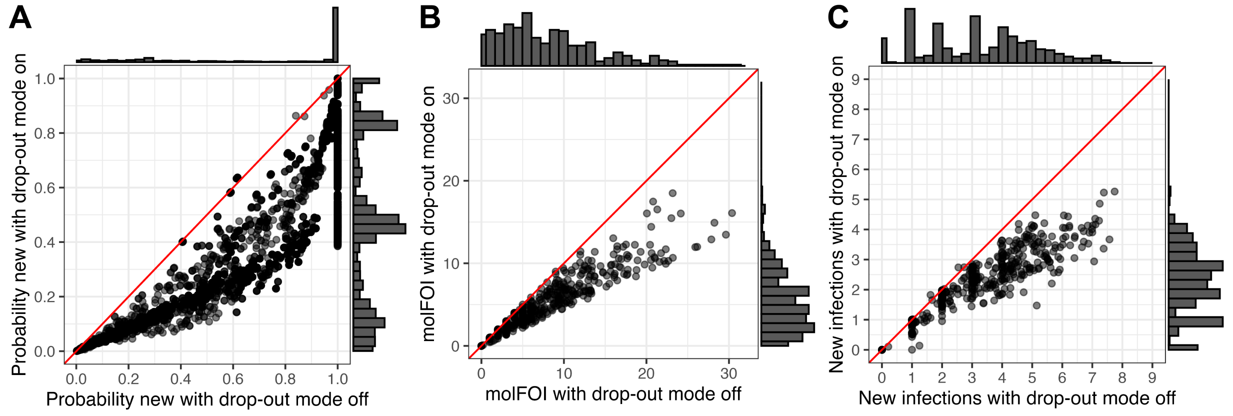

Supplementary Figure 9. A Bayesian model allowing for newly observed alleles to not be counted as new due to sequencing drop out produced lower estimates of the probability an individual sequenced allele was new (A), the per-subject molFOI (B), and the per-subject number of infection events (C). For the 2011 Malian cohort, the same model parameters as described in the **Methods** were used but with the drop-out model described in the **Supplementary Information**.
